# Prevention of mother-to-child transmission activities after one-off clinical mentorship training in selected health facilities, Zimbabwe: 2014-2018

**DOI:** 10.11604/pamj.2020.36.146.19542

**Published:** 2020-07-01

**Authors:** Winnie Mandewo, Cephas Muchuchuti, Obey Shoko, Collins Timire, Kudakwashe Collin Takarinda, Anthony David Harries, Hannock Tweya, Talent Tapera, Saziso Nyathi, Addmore Chadambuka, Anesu Chimwaza, Agnes Mahomva

**Affiliations:** 1 Elizabeth Glaser Pediatric AIDS Foundation (EGPAF), Harare, Zimbabwe,; 2 International Union Against Tuberculosis and Lung Disease, Harare, Zimbabwe,; 3 International Union Against Tuberculosis and Lung Disease, Paris, France,; 4 AIDS & TB Department, Ministry of Health and Child Care, Harare, Zimbabwe,; 5 London School of Hygiene and Tropical Medicine, London, UK,; 6 The Lighthouse Trust, Lilongwe, Malawi,; 7 Africaid Zvandiri, Avondale, Harare, Zimbabwe,; 8 Health Services Department, City of Bulawayo, Zimbabwe

**Keywords:** Elizabeth Glaser Paediatric AIDS Foundation (EGPAF), HIV-infected pregnant women, HIV-exposed infants, early infant diagnosis, antiretroviral therapy

## Abstract

This was a cross-sectional study describing HIV testing uptake and ART initiation for pregnant women and HIV-exposed infants after one-off clinical mentorship training in 2013 for nurses in 56 peripheral health-facilities, Zimbabwe. Between 2014-2018, 92% of 106411 pregnant women were HIV tested and 98% of HIV-positive women initiated antiretroviral therapy (ART). There were 15846 HIV-exposed infants, of whom 96% had dried blood spots collected for virologic diagnosis and 51% of those diagnosed HIV-positive initiated ART. In conclusion, this one-off clinical mentorship training in 2013 was associated with consistently high HIV testing and ART initiation in pregnant women and their children.

## Introduction

Despite good global progress in the prevention of mother-to-child transmission of HIV (PMTCT), implementation is still inadequate with an estimated 180,000 new HIV infections in children and just 52% of HIV-infected children accessing antiretroviral therapy (ART) in 2017 [1]. Most of these infections occur in sub-Saharan Africa, particularly high HIV-burden countries like Zimbabwe. In 2013, Zimbabwe adopted option B+ [all HIV-infected pregnant women start life-long ART regardless of immune-suppression] [2], which was accompanied by a policy of task-shifting from doctor-led to nurse-led ART initiation coupled with decentralisation of ART services to primary health-care facilities. The Elizabeth Glaser Pediatric AIDS Foundation (EGPAF) supported Zimbabwe in this approach through a one-off clinical mentorship training programme for nurses in 2013 involving in-class training and clinical attachments. In those facilities, ART initiation in pregnant women increased from 25% before mentorship training in 2012 to 67% after clinical mentorship training in 2013 [3]. We were interested to know whether these good results were maintained in the following four years. This study therefore aimed to describe what happened to HIV testing uptake and ART initiation for pregnant women and their HIV-exposed infants after this mentorship training in 56 predominately rural health facilities in Zimbabwe between 2014 and 2018.

## Methods

This was a cross-sectional study using routinely collected secondary aggregate data. The clinical mentorship training for nurses included five-days of classroom training by clinicians and nurses, seven-days when the nurse was attached to a clinician involved in ART care and four-months of supervision from a clinical mentor and multidisciplinary team observing nurse´s practices in HIV and ART management and care [3]. EGPAF organized and implemented the mentorship training which was rolled out in a phased approach to health facilities in seven provinces, with 1-4 nurses being trained in each facility. Following the training, nurses offered pregnant women HIV testing and counselling and initiated ART in those diagnosed HIV-positive. They also collected dried blood spot (DBS) samples from HIV-exposed children 6 weeks after birth and initiated ART in children diagnosed HIV-positive through DNA-PCR testing at the central laboratories. Data were captured in paper-based registers with aggregate monthly data uploaded to the government DHIS-2 (District Health Information Systems, Version 2.0) electronic database. For the study, EGPAF randomly selected 56 health-facilities (49 rural health-centers, 2 urban health-centers and 5 rural hospitals) in which 146 nurses had received training. The study population included pregnant women enrolled in antenatal care and HIV-exposed children born to HIV-positive mothers in these health facilities from 2014 to 2018. Data variables were downloaded from the DHIS-2 electronic database and analyzed in Excel. A descriptive analysis was performed. Comparisons of HIV testing and ART initiation in relation to urban versus rural facilities and to numbers of nurses trained were undertaken using Pearson´s Chi-square test for aggregate data. Levels of significance were set at 5% (P < 0.05). Ethics approval was obtained from the medical research council, Zimbabwe and the union ethics advisory group, Paris, France.

## Results

From 2014-2018, there were 106411 pregnant women enrolled in antenatal care, 97829 (92%) were HIV tested, 6097 (6%) were HIV-positive and 6004 (98%) were started on ART. Annual HIV testing uptake ranged from 91%-93%. ART initiation in those diagnosed HIV-positive ranged from 97%-99%. Characteristics associated with HIV testing uptake and ART initiation are shown in Table 1. Key findings were higher HIV testing uptake and ART initiation in urban compared with rural health-centers and rural hospitals. From 2014-2018, there were 15846 HIV-exposed infants (from mothers newly diagnosed HIV-positive and initiated on ART and mothers already known to be HIV-positive and on ART), 15235 (96%) had DBS collected for DNA-PCR testing, 1926 (13%) infants were diagnosed HIV-positive and 980 (51%) were started on ART. Annual DBS collection in HIV-exposed infants ranged from 95%-99% and ART initiation in HIV-positive infants ranged from 45% to 61% with no consistent trend shown over five years. Characteristics associated with DBS collection and ART initiation are shown in Table 2. Key findings were higher DBS collections in urban health centers compared with rural health centers/rural hospitals and in health facilities with 4 nurses trained compared with 1-2 nurses trained.

**Table 1:** characteristics associated with uptake of HIV testing and initiation on antiretroviral therapy in pregnant women enrolled in antenatal care in EGPAF-supported health facilities, Zimbabwe between 2014 and 2018

Characteristics	Pregnant women					
	Enrolled	HIV tested		HIV-positive	Initiated on ART	
	n	n	(%)	n	n	(%)
Total	106411	97829	(92)	6097	6004	(98)
**Type of health facility:**						
Urban Health Center	4401	4185	(95)	294	294	(100)
Rural Health Center	91206	83806	(92)^a^	5276	5190	(98)^b^
Rural Hospital	10804	9838	(91)^a^	527	520	(99)
**Nurses trained per facility:**						
One	4794	4416	(92)	297	296	(99)
Two	66187	61143	(92)	3472	3381	(97)^c^
Three	18336	16514	(90)^c^	1297	1296	(99)
Four	17094	15756	(92)	1031	1031	(100)

EGPAF=Elizabeth Glaser Pediatric AIDS Foundation; ART=antiretroviral therapy; ^a^P <0.001 and ^b^P <0.05-compared with urban health centers; ^c^P <0.001-compared with facilities that had four nurses trained

**Table 2:** characteristics associated with collection of dried blood spots for HIV-DNA PCR testing and initiation of antiretroviral therapy in HIV-exposed infants in EGPAF-supported health facilities, Zimbabwe between 2014 and 2018

Characteristics	HIV-exposed infants born to HIV-positive mothers					
	HIV-exposed	DBS collected		HIV-positive	Initiated on ART	
	n	n	(%)	n	n	(%)
Total	15846	15235	(96)	1926	980	(51)
**Type of health facility:**						
Urban Health Center	1424	1424	(100)	197	95	(48)
Rural Health Center	13669	13176	(96)^a^	1622	827	(51)
Rural Hospital	753	635	(84)^a^	107	58	(54)
**Nurses trained per facility:**						
One	106	92	(87)^b^	7	7	(100)^c^
Two	11146	10631	(95)^b^	1339	701	(52)
Three	2386	2349	(98)	293	133	(45)
Four	2208	2163	(98)	287	139	(48)

EGPAF=Elizabeth Glaser Pediatric AIDS Foundation; DBS=dried blood spots for HIV-DNA PCR testing; ART=antiretroviral therapy; ^a^P <0.001-compared with urban health centers; ^b^P <0.001 and ^c^P <0.05-compared with facilities that had four nurses trained

## Discussion

Following the clinical mentorship training in 2013, annual HIV testing uptake in pregnant women was consistently high >90% and ART initiation in HIV-positive women was also consistently high >95%. These findings align with national reports, [4] and compare favorably with recent reports on PMTCT in other African countries [5]. The inferior findings from rural facilities may be due to difficult access to PMTCT services and infrastructure issues [6]. For HIV-exposed infants, 6-weeks DBS collections were consistently >95%, with performance better in urban facilities and facilities with more nurses trained. These findings also align with national reports [4]. The one negative finding was poor ART coverage for HIV-infected infants, with no differences found between urban and rural facilities or related to numbers of nurses trained. Similar findings on ART have been reported from other predominately rural settings in Africa [7]. One finding that deserves comment was the high HIV-infection rate in HIV-exposed infants compared with national estimates [4].

This may be due to our use of aggregate rather than individual cascade data, possible data documentation errors or a true reflection of MTCT in rural Zimbabwe. A recent review in Ethiopia showed that while the pooled MTCT prevalence was 10%, this ranged from 5-15% in different parts of the country [8]. Study strengths included the large number of health facilities and high numbers of pregnant women and HIV-exposed children. There were a number of limitations. Use of aggregate data prevented us from more precisely describing the cascade of care for mothers and their HIV-exposed children. We also had no information about how many mothers were on established ART at the time of enrolment at the clinics, the timing of events or details about relevant laboratory investigations such as CD4 cell counts or ART regimens. There are two main lessons. First, the one-off clinical mentorship programme for nurses in 2013 resulted in generally good HIV testing and ART uptake in the following five years. While we cannot attribute cause and effect, EGPAF should consider further one-off clinical mentorship training for better implementation of new PMTCT strategies and practices [9]. Second, peripheral health-facilities should consider moving to electronic data-capture systems in order to better monitor the UNAIDS 90-90-90 cascade targets [10].

## Conclusion

After one-off clinical mentorship training for nurses on PMTCT in 56 health-facilities in Zimbabwe, there was consistent high uptake of HIV testing and ART initiation in pregnant women and collection of DBS in HIV-exposed children. However, initiation of ART in children was sub-optimal. With changing PMTCT practices in Zimbabwe and a move from paper-based to electronic data-capture systems further clinical mentorship training for nurses should be considered.

### What is known about this topic

- Clinical mentorship program involving a change from doctor-led to nurse-led task shifting is feasible and results in improvement in HIV testing uptake and ART initiation for both pregnant mothers and their HIV exposed babies;
- Nurse led-ART has been shown to improve both HIV testing uptake and ART initiation of pregnant women and their HIV positive babies.

### What this study adds

- A one-off clinical mentorship programme has the potential to maintain consistently high annual HIV testing (at > 90%) and ART initiation in HIV positive pregnant mothers (at >95%) even three to four years post mentorship;
- Both accessibility to health facilities and dose-responses to DBS uptake and ART initiation were observed as urban facilities and facilities with at least one trained nurses showed a higher performance of DBS uptake and ART initiation.
